# Crystal structure of (*E*)-2-(4-meth­oxy­styr­yl)-3-methyl-1-phenyl­sulfonyl-1*H*-indole

**DOI:** 10.1107/S2056989015016631

**Published:** 2015-09-12

**Authors:** M. Umadevi, P. Raju, R. Yamuna, A. K. Mohanakrishnan, G. Chakkaravarthi

**Affiliations:** a Research and Development Centre, Bharathiar University, Coimbatore 641 046, India; b Department of Chemistry, Pallavan College of Engineering, Kanchipuram 631 502, India; c Department of Organic Chemistry, University of Madras, Guindy Campus, Chennai 600 025, India; d Department of Sciences, Chemistry and Materials Research Lab, Amrita Vishwa Vidyapeetham University, Ettimadai, Coimbatore 641 112, India; e Department of Physics, CPCL Polytechnic College, Chennai 600 068, India

**Keywords:** crystal structure, phenyl­sulfon­yl, 1*H*-indole, hydrogen bonding, C—H⋯π inter­actions, π–π inter­actions

## Abstract

In the title compound, C_24_H_21_NO_3_S, the dihedral angles between the indole ring system (r.m.s. deviation = 0.030 Å) and the sulfur and ethyl­ene-bonded benzene rings are 80.2 (2) and 49.29 (15)°, respectively. The dihedral angle between the pendant benzene rings is 37.7 (2)°. In the crystal, mol­ecules are linked by C—H⋯O hydrogen bonds and weak C—H⋯π and π–π [centroid-to-centroid distances = 3.549 (2) and 3.743 (3) Å] inter­actions, forming a three-dimensional network.

## Related literature   

For the biological activity of indole derivatives, see: Andreani *et al.* (2001 ▸); Kolocouris *et al.* (1994 ▸). For the structures of related compounds, see: Chakkaravarthi *et al.* (2007 ▸, 2008 ▸).
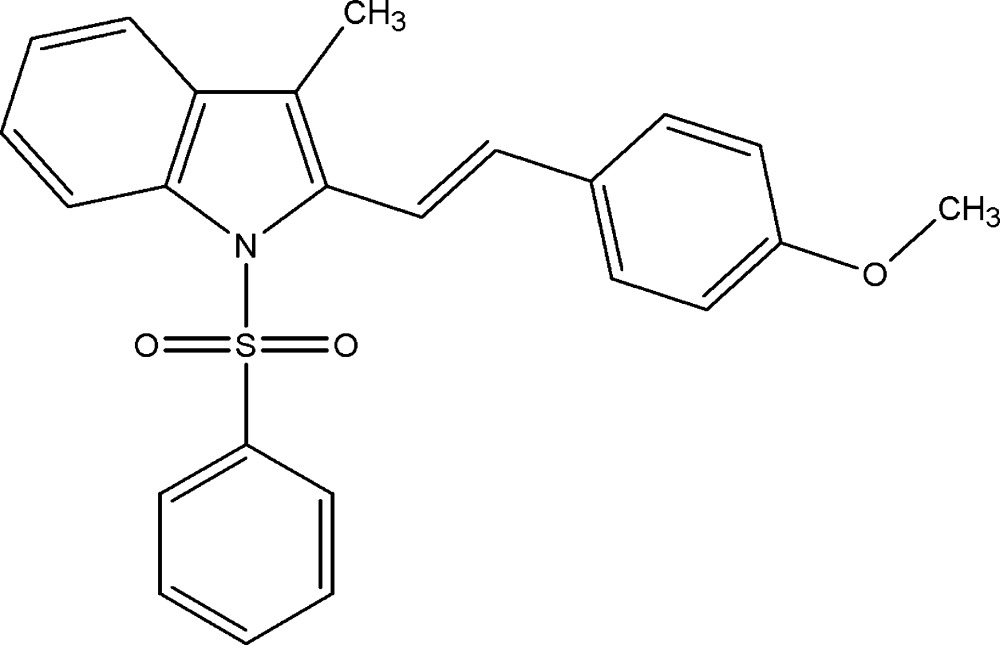


## Experimental   

### Crystal data  

C_24_H_21_NO_3_S
*M* *_r_* = 403.48
Monoclinic, 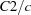

*a* = 27.373 (4) Å
*b* = 12.7232 (16) Å
*c* = 12.0881 (13) Å
β = 102.827 (6)°
*V* = 4104.9 (9) Å^3^
*Z* = 8
Mo *K*α radiation
μ = 0.18 mm^−1^
*T* = 295 K
0.28 × 0.24 × 0.20 mm

### Data collection  

Bruker Kappa APEXII CCD diffractometer
Absorption correction: multi-scan (*SADABS*; Sheldrick, 1996 ▸) *T*_min_ = 0.951, *T*_max_ = 0.964
26597 measured reflections
4852 independent reflections
2432 reflections with *I* > 2σ(*I*)
*R*_int_ = 0.120

### Refinement  

*R*[*F* ^2^ > 2σ(*F* ^2^)] = 0.064
*wR*(*F* ^2^) = 0.231
*S* = 1.00
4852 reflections
264 parameters
H-atom parameters constrained
Δρ_max_ = 0.28 e Å^−3^
Δρ_min_ = −0.37 e Å^−3^

### 

Data collection: *APEX2* (Bruker, 2004 ▸); cell refinement: *SAINT* (Bruker, 2004 ▸); data reduction: *SAINT*; program(s) used to solve structure: *SHELXS97* (Sheldrick, 2008 ▸); program(s) used to refine structure: *SHELXL97* (Sheldrick, 2008 ▸); molecular graphics: *PLATON* (Spek, 2009 ▸); software used to prepare material for publication: *SHELXL97* and *PLATON*.

## Supplementary Material

Crystal structure: contains datablock(s) global, I. DOI: 10.1107/S2056989015016631/hb7496sup1.cif

Structure factors: contains datablock(s) I. DOI: 10.1107/S2056989015016631/hb7496Isup2.hkl

Supporting information file. DOI: 10.1107/S2056989015016631/hb7496Isup3.cml

**Fig. 1.**
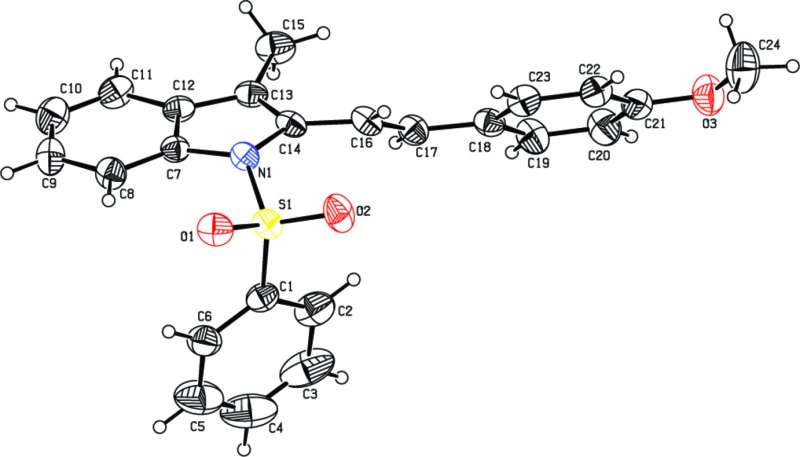
The molecular structure of (I), with 30% probability displacement ellipsoids for non-H atoms.

**Fig. 2.**
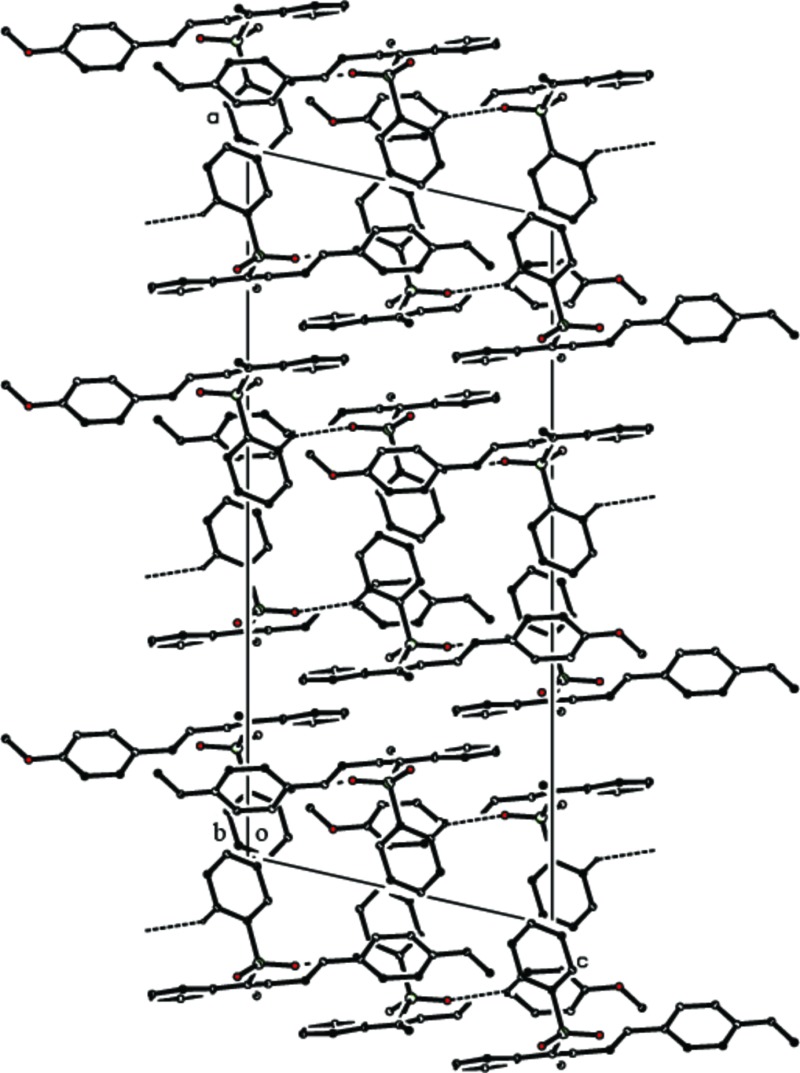
The crystal packing of the title compound viewed along the b axis. The hydrogen bonds are shown as dashed lines (see Table 1), and C-bound H atoms have been omitted for clarity.

. DOI: 10.1107/S2056989015016631/hb7496fig1.tif
The mol­ecular structure of (I), with 30% probability displacement ellipsoids for non-H atoms.

b . DOI: 10.1107/S2056989015016631/hb7496fig2.tif
The crystal packing of the title compound viewed along the *b* axis. The hydrogen bonds are shown as dashed lines (see Table 1), and C-bound H atoms have been omitted for clarity.

CCDC reference: 1422542

Additional supporting information:  crystallographic information; 3D view; checkCIF report

## Figures and Tables

**Table 1:** Hydrogen-bond geometry (, ) *Cg*2, *Cg*3 and *Cg*4 are the centroids of the C1C6, C7C12 and C18C23 rings, respectively.

*D*H*A*	*D*H	H*A*	*D* *A*	*D*H*A*
C6H6O2^i^	0.93	2.46	3.249(5)	143
C15H15*C* *Cg*4^ii^	0.96	2.82	3.759(4)	167
C24H24*A* *Cg*3^iii^	0.96	2.84	3.634(6)	140
C24H24*C* *Cg*2^iii^	0.96	2.88	3.520(5)	125

Symmetry codes: (i) 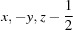; (ii) 
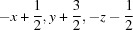
; (iii) 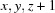.

## supplementary crystallographic information

### S1. Comment

Indole derivatives exhibit antitumour (Andreani *et al.*, 2001) and antiviral (Kolocouris *et al.*, 1994) activities. The molecular structure of the title compound is illustrated in Fig. 1. The geometric parameters of the title molecule agree well with the reported similar structures (Chakkaravarthi *et al.* 2007, 2008). The torsion angles O1—S1—N1—C7 and O2—S1—N1—C14 [-48.0 (3)° and 38.3 (3)°, respectively] indicate the *syn*-conformation of the sulfonyl moiety.

In the crystal, the molecules are linked by C—H···O hydrogen bonds (Table 1 & Fig. 2) and the packing also features weak C—H···π (Table 1) and π–π [*Cg*1···*Cg*1^i^ distance 3.549 (2) Å; *Cg*2···*Cg*2^ii^ distance 3.743 (3) Å; (i) 1/2 - *x*,1/2 - *y*,1 - *z*; 1 - *x*,-*y*,1 - *z*; *Cg*1 and *Cg*2 are the centroids of the rings (N1/C7/C12/C13/C14) and (C1—C6), respectively] interactions in a three-dimensional network.

### S2. Experimental

To a suspension of sodium hydride (0.22 g, 4.74 mmol) in dry THF (10 ml) at -10°C, the solution of diethyl (3-methyl-1-(phenylsulfonyl)-1*H*-indol-2-yl)methylphosphonate (1 g, 2.37 mmol) in dry THF (10 ml) was slowly added under nitrogen atmosphere and stirred for 1 h at -10°C. Then, the solution of *p*-anisaldehyde (0.31 ml, 2.61 mmol) in dry THF (5 ml) was added and the stirring was continued at -10 to 0°C for another 2 h. After completion of the reaction (monitored by TLC), the yellow solution was poured over crushed ice (80 g) containing Conc. HCl (5 ml). The solid obtained was filtered, dried and recrystallized from methanol solution to afford the title compound in the form of colourless blocks.

### S3. Refinement

H atoms were positioned geometrically and refined using riding model, with C—H = 0.93 Å and *U*_iso_(H) = 1.2Ueq(C) for C—H and C—H = 0.96 Å and *U*_iso_(H) = 1.5Ueq(C) for methyl. The reflections (2 0 0) and (1 1 0) were omitted during refinement which were owing poor agreement.

### Crystal data

**Table:**

C_24_H_21_NO_3_S	*F*(000) = 1696
*M**_r_* = 403.48	*D*_x_ = 1.306 Mg m^−^^3^
Monoclinic, *C*2/*c*	Mo *K*α radiation, λ = 0.71073 Å
Hall symbol: -C 2yc	Cell parameters from 5639 reflections
*a* = 27.373 (4) Å	θ = 2.6–24.6°
*b* = 12.7232 (16) Å	µ = 0.18 mm^−^^1^
*c* = 12.0881 (13) Å	*T* = 295 K
β = 102.827 (6)°	Block, colourless
*V* = 4104.9 (9) Å^3^	0.28 × 0.24 × 0.20 mm
*Z* = 8	

### Data collection

**Table:**

Bruker Kappa APEXII CCD diffractometer	4852 independent reflections
Radiation source: fine-focus sealed tube	2432 reflections with *I* > 2σ(*I*)
Graphite monochromator	*R*_int_ = 0.120
ω and φ scan	θ_max_ = 27.9°, θ_min_ = 2.4°
Absorption correction: multi-scan (*SADABS*; Sheldrick, 1996)	*h* = −36→35
*T*_min_ = 0.951, *T*_max_ = 0.964	*k* = −16→16
26597 measured reflections	*l* = −15→15

### Refinement

**Table:**

Refinement on *F*^2^	Primary atom site location: structure-invariant direct methods
Least-squares matrix: full	Secondary atom site location: difference Fourier map
*R*[*F*^2^ > 2σ(*F*^2^)] = 0.064	Hydrogen site location: inferred from neighbouring sites
*wR*(*F*^2^) = 0.231	H-atom parameters constrained
*S* = 1.00	*w* = 1/[σ^2^(*F*_o_^2^) + (0.109*P*)^2^ + 3.4059*P*] where *P* = (*F*_o_^2^ + 2*F*_c_^2^)/3
4852 reflections	(Δ/σ)_max_ < 0.001
264 parameters	Δρ_max_ = 0.28 e Å^−^^3^
0 restraints	Δρ_min_ = −0.37 e Å^−^^3^

### Special details

**Table:**

Geometry. All e.s.d.'s (except the e.s.d. in the dihedral angle between two l.s. planes) are estimated using the full covariance matrix. The cell e.s.d.'s are taken into account individually in the estimation of e.s.d.'s in distances, angles and torsion angles; correlations between e.s.d.'s in cell parameters are only used when they are defined by crystal symmetry. An approximate (isotropic) treatment of cell e.s.d.'s is used for estimating e.s.d.'s involving l.s. planes.
Refinement. Refinement of *F*^2^ against ALL reflections. The weighted *R*-factor *wR* and goodness of fit *S* are based on *F*^2^, conventional *R*-factors *R* are based on *F*, with *F* set to zero for negative *F*^2^. The threshold expression of *F*^2^ > σ(*F*^2^) is used only for calculating *R*-factors(gt) *etc*. and is not relevant to the choice of reflections for refinement. *R*-factors based on *F*^2^ are statistically about twice as large as those based on *F*, and *R*- factors based on ALL data will be even larger.

### Fractional atomic coordinates and isotropic or equivalent isotropic displacement parameters (Å^2^)

**Table:**

	*x*	*y*	*z*	*U*_iso_*/*U*_eq_	
C1	0.40832 (12)	0.0851 (3)	0.4987 (3)	0.0558 (9)	
C2	0.44297 (16)	0.1492 (3)	0.5659 (4)	0.0830 (12)	
H2	0.4371	0.1778	0.6326	0.100*	
C3	0.48806 (19)	0.1704 (4)	0.5304 (7)	0.117 (2)	
H3	0.5124	0.2133	0.5741	0.140*	
C4	0.4957 (2)	0.1274 (5)	0.4313 (7)	0.121 (2)	
H4	0.5254	0.1412	0.4083	0.145*	
C5	0.4609 (2)	0.0657 (5)	0.3678 (5)	0.1072 (18)	
H5	0.4667	0.0369	0.3011	0.129*	
C6	0.41692 (15)	0.0444 (3)	0.3994 (3)	0.0697 (10)	
H6	0.3928	0.0024	0.3536	0.084*	
C7	0.30101 (11)	0.1982 (2)	0.3861 (3)	0.0467 (7)	
C8	0.29070 (13)	0.1401 (3)	0.2867 (3)	0.0583 (9)	
H8	0.2952	0.0676	0.2882	0.070*	
C9	0.27364 (14)	0.1922 (4)	0.1857 (3)	0.0712 (11)	
H9	0.2671	0.1542	0.1183	0.085*	
C10	0.26607 (15)	0.2987 (4)	0.1820 (3)	0.0748 (11)	
H10	0.2537	0.3314	0.1126	0.090*	
C11	0.27647 (13)	0.3574 (3)	0.2794 (3)	0.0642 (10)	
H11	0.2713	0.4297	0.2761	0.077*	
C12	0.29493 (11)	0.3081 (3)	0.3841 (3)	0.0496 (8)	
C13	0.31014 (12)	0.3465 (2)	0.4972 (3)	0.0516 (8)	
C14	0.32558 (11)	0.2641 (2)	0.5673 (3)	0.0480 (8)	
C15	0.30517 (15)	0.4586 (3)	0.5297 (4)	0.0736 (11)	
H15A	0.3076	0.4632	0.6100	0.110*	
H15B	0.2732	0.4853	0.4902	0.110*	
H15C	0.3314	0.4994	0.5097	0.110*	
C16	0.34235 (13)	0.2629 (3)	0.6895 (3)	0.0537 (8)	
H16	0.3298	0.2110	0.7298	0.064*	
C17	0.37458 (13)	0.3316 (3)	0.7474 (3)	0.0556 (8)	
H17	0.3887	0.3794	0.7054	0.067*	
C18	0.38995 (12)	0.3393 (3)	0.8702 (3)	0.0534 (8)	
C19	0.42576 (14)	0.4133 (3)	0.9200 (3)	0.0675 (10)	
H19	0.4405	0.4553	0.8736	0.081*	
C20	0.43979 (15)	0.4256 (3)	1.0354 (3)	0.0734 (11)	
H20	0.4635	0.4762	1.0657	0.088*	
C21	0.41924 (13)	0.3642 (3)	1.1070 (3)	0.0619 (9)	
C22	0.38457 (12)	0.2888 (3)	1.0604 (3)	0.0589 (9)	
H22	0.3707	0.2457	1.1075	0.071*	
C23	0.37042 (13)	0.2769 (3)	0.9445 (3)	0.0602 (9)	
H23	0.3470	0.2255	0.9148	0.072*	
C24	0.4123 (2)	0.3287 (4)	1.2952 (4)	0.1073 (17)	
H24A	0.3772	0.3454	1.2805	0.161*	
H24B	0.4278	0.3481	1.3717	0.161*	
H24C	0.4164	0.2547	1.2852	0.161*	
N1	0.31802 (9)	0.16882 (19)	0.5006 (2)	0.0477 (7)	
O1	0.32587 (9)	−0.02048 (17)	0.4661 (2)	0.0633 (7)	
O2	0.35975 (11)	0.0507 (2)	0.65544 (19)	0.0733 (8)	
O3	0.43465 (10)	0.3842 (3)	1.2199 (2)	0.0862 (9)	
S1	0.35144 (3)	0.06038 (6)	0.53598 (7)	0.0512 (3)	

### Atomic displacement parameters (Å^2^)

**Table:**

	*U*^11^	*U*^22^	*U*^33^	*U*^12^	*U*^13^	*U*^23^
C1	0.0529 (19)	0.0443 (19)	0.068 (2)	0.0044 (15)	0.0089 (17)	0.0051 (16)
C2	0.071 (3)	0.058 (3)	0.112 (3)	0.002 (2)	0.005 (2)	−0.013 (2)
C3	0.064 (3)	0.071 (3)	0.204 (7)	−0.011 (2)	0.008 (4)	0.002 (4)
C4	0.082 (4)	0.097 (4)	0.199 (7)	0.008 (3)	0.065 (4)	0.028 (4)
C5	0.088 (3)	0.114 (4)	0.138 (5)	0.007 (3)	0.064 (3)	0.021 (4)
C6	0.066 (2)	0.076 (3)	0.071 (3)	0.0089 (19)	0.0230 (19)	0.004 (2)
C7	0.0452 (17)	0.0462 (19)	0.0502 (19)	0.0007 (14)	0.0141 (14)	0.0035 (15)
C8	0.065 (2)	0.048 (2)	0.061 (2)	−0.0047 (16)	0.0105 (17)	−0.0074 (17)
C9	0.078 (3)	0.081 (3)	0.051 (2)	−0.007 (2)	0.0066 (19)	0.001 (2)
C10	0.079 (3)	0.074 (3)	0.068 (3)	−0.003 (2)	0.009 (2)	0.017 (2)
C11	0.063 (2)	0.053 (2)	0.076 (3)	0.0011 (17)	0.0119 (19)	0.016 (2)
C12	0.0437 (17)	0.0437 (19)	0.063 (2)	0.0011 (14)	0.0156 (15)	0.0046 (16)
C13	0.0476 (18)	0.0409 (19)	0.069 (2)	0.0003 (14)	0.0194 (16)	−0.0030 (17)
C14	0.0498 (18)	0.0418 (18)	0.057 (2)	−0.0044 (14)	0.0227 (15)	−0.0097 (15)
C15	0.075 (3)	0.044 (2)	0.102 (3)	0.0026 (18)	0.021 (2)	−0.008 (2)
C16	0.063 (2)	0.047 (2)	0.057 (2)	−0.0032 (16)	0.0262 (17)	−0.0063 (15)
C17	0.059 (2)	0.053 (2)	0.056 (2)	−0.0041 (16)	0.0173 (16)	−0.0039 (16)
C18	0.0542 (19)	0.046 (2)	0.062 (2)	−0.0012 (15)	0.0176 (16)	−0.0080 (16)
C19	0.074 (2)	0.067 (2)	0.066 (2)	−0.018 (2)	0.026 (2)	−0.0078 (19)
C20	0.068 (2)	0.079 (3)	0.074 (3)	−0.025 (2)	0.018 (2)	−0.019 (2)
C21	0.057 (2)	0.066 (2)	0.062 (2)	0.0018 (18)	0.0109 (18)	−0.0069 (19)
C22	0.057 (2)	0.057 (2)	0.063 (2)	−0.0004 (17)	0.0147 (17)	0.0087 (18)
C23	0.058 (2)	0.050 (2)	0.071 (2)	−0.0051 (16)	0.0119 (18)	−0.0014 (18)
C24	0.148 (5)	0.098 (4)	0.067 (3)	−0.013 (3)	0.005 (3)	0.018 (3)
N1	0.0532 (15)	0.0406 (15)	0.0509 (16)	−0.0033 (12)	0.0149 (12)	−0.0032 (12)
O1	0.0731 (16)	0.0360 (13)	0.0815 (17)	−0.0048 (11)	0.0189 (13)	−0.0054 (11)
O2	0.113 (2)	0.0603 (16)	0.0497 (15)	0.0094 (14)	0.0244 (14)	0.0132 (12)
O3	0.0832 (19)	0.109 (2)	0.0646 (18)	−0.0207 (17)	0.0131 (15)	−0.0117 (16)
S1	0.0654 (6)	0.0367 (5)	0.0536 (5)	0.0005 (4)	0.0176 (4)	0.0033 (4)

### Geometric parameters (Å, º)

**Table:**

C1—C2	1.372 (5)	C14—C16	1.447 (4)
C1—C6	1.375 (5)	C15—H15A	0.9600
C1—S1	1.742 (4)	C15—H15B	0.9600
C2—C3	1.420 (7)	C15—H15C	0.9600
C2—H2	0.9300	C16—C17	1.326 (4)
C3—C4	1.375 (8)	C16—H16	0.9300
C3—H3	0.9300	C17—C18	1.454 (5)
C4—C5	1.337 (8)	C17—H17	0.9300
C4—H4	0.9300	C18—C23	1.391 (5)
C5—C6	1.369 (6)	C18—C19	1.395 (5)
C5—H5	0.9300	C19—C20	1.372 (5)
C6—H6	0.9300	C19—H19	0.9300
C7—C8	1.386 (4)	C20—C21	1.376 (5)
C7—C12	1.407 (5)	C20—H20	0.9300
C7—N1	1.409 (4)	C21—O3	1.359 (4)
C8—C9	1.377 (5)	C21—C22	1.379 (5)
C8—H8	0.9300	C22—C23	1.377 (5)
C9—C10	1.370 (6)	C22—H22	0.9300
C9—H9	0.9300	C23—H23	0.9300
C10—C11	1.370 (5)	C24—O3	1.396 (5)
C10—H10	0.9300	C24—H24A	0.9600
C11—C12	1.403 (5)	C24—H24B	0.9600
C11—H11	0.9300	C24—H24C	0.9600
C12—C13	1.424 (5)	N1—S1	1.658 (3)
C13—C14	1.355 (4)	O1—S1	1.414 (2)
C13—C15	1.494 (5)	O2—S1	1.416 (2)
C14—N1	1.446 (4)		
			
C2—C1—C6	120.7 (4)	H15A—C15—H15B	109.5
C2—C1—S1	119.6 (3)	C13—C15—H15C	109.5
C6—C1—S1	119.6 (3)	H15A—C15—H15C	109.5
C1—C2—C3	117.8 (5)	H15B—C15—H15C	109.5
C1—C2—H2	121.1	C17—C16—C14	123.7 (3)
C3—C2—H2	121.1	C17—C16—H16	118.2
C4—C3—C2	120.0 (5)	C14—C16—H16	118.2
C4—C3—H3	120.0	C16—C17—C18	126.3 (3)
C2—C3—H3	120.0	C16—C17—H17	116.9
C5—C4—C3	120.4 (5)	C18—C17—H17	116.9
C5—C4—H4	119.8	C23—C18—C19	116.1 (3)
C3—C4—H4	119.8	C23—C18—C17	123.7 (3)
C4—C5—C6	121.0 (5)	C19—C18—C17	120.2 (3)
C4—C5—H5	119.5	C20—C19—C18	121.8 (3)
C6—C5—H5	119.5	C20—C19—H19	119.1
C5—C6—C1	120.1 (5)	C18—C19—H19	119.1
C5—C6—H6	120.0	C19—C20—C21	120.9 (4)
C1—C6—H6	120.0	C19—C20—H20	119.5
C8—C7—C12	121.0 (3)	C21—C20—H20	119.5
C8—C7—N1	132.0 (3)	O3—C21—C20	116.5 (3)
C12—C7—N1	107.0 (3)	O3—C21—C22	124.9 (3)
C9—C8—C7	118.4 (3)	C20—C21—C22	118.6 (3)
C9—C8—H8	120.8	C23—C22—C21	120.3 (3)
C7—C8—H8	120.8	C23—C22—H22	119.9
C10—C9—C8	121.6 (4)	C21—C22—H22	119.9
C10—C9—H9	119.2	C22—C23—C18	122.2 (3)
C8—C9—H9	119.2	C22—C23—H23	118.9
C9—C10—C11	120.7 (4)	C18—C23—H23	118.9
C9—C10—H10	119.7	O3—C24—H24A	109.5
C11—C10—H10	119.7	O3—C24—H24B	109.5
C10—C11—C12	119.7 (4)	H24A—C24—H24B	109.5
C10—C11—H11	120.2	O3—C24—H24C	109.5
C12—C11—H11	120.2	H24A—C24—H24C	109.5
C11—C12—C7	118.6 (3)	H24B—C24—H24C	109.5
C11—C12—C13	133.0 (3)	C7—N1—C14	107.5 (2)
C7—C12—C13	108.4 (3)	C7—N1—S1	121.2 (2)
C14—C13—C12	108.6 (3)	C14—N1—S1	123.5 (2)
C14—C13—C15	127.4 (3)	C21—O3—C24	118.4 (3)
C12—C13—C15	123.8 (3)	O1—S1—O2	119.45 (15)
C13—C14—N1	108.3 (3)	O1—S1—N1	106.24 (14)
C13—C14—C16	129.2 (3)	O2—S1—N1	106.86 (14)
N1—C14—C16	122.3 (3)	O1—S1—C1	109.21 (16)
C13—C15—H15A	109.5	O2—S1—C1	109.19 (17)
C13—C15—H15B	109.5	N1—S1—C1	104.87 (14)
			
C6—C1—C2—C3	−1.1 (6)	C23—C18—C19—C20	−1.8 (5)
S1—C1—C2—C3	−177.7 (3)	C17—C18—C19—C20	177.5 (3)
C1—C2—C3—C4	0.2 (7)	C18—C19—C20—C21	0.7 (6)
C2—C3—C4—C5	0.2 (9)	C19—C20—C21—O3	−178.4 (4)
C3—C4—C5—C6	0.3 (9)	C19—C20—C21—C22	0.8 (6)
C4—C5—C6—C1	−1.1 (7)	O3—C21—C22—C23	178.1 (3)
C2—C1—C6—C5	1.5 (6)	C20—C21—C22—C23	−1.1 (5)
S1—C1—C6—C5	178.2 (3)	C21—C22—C23—C18	−0.1 (5)
C12—C7—C8—C9	−0.9 (5)	C19—C18—C23—C22	1.5 (5)
N1—C7—C8—C9	179.2 (3)	C17—C18—C23—C22	−177.7 (3)
C7—C8—C9—C10	−1.0 (6)	C8—C7—N1—C14	175.9 (3)
C8—C9—C10—C11	1.6 (6)	C12—C7—N1—C14	−4.0 (3)
C9—C10—C11—C12	−0.2 (6)	C8—C7—N1—S1	25.9 (4)
C10—C11—C12—C7	−1.7 (5)	C12—C7—N1—S1	−154.0 (2)
C10—C11—C12—C13	178.2 (3)	C13—C14—N1—C7	4.5 (3)
C8—C7—C12—C11	2.2 (5)	C16—C14—N1—C7	179.0 (3)
N1—C7—C12—C11	−177.8 (3)	C13—C14—N1—S1	153.6 (2)
C8—C7—C12—C13	−177.7 (3)	C16—C14—N1—S1	−31.8 (4)
N1—C7—C12—C13	2.2 (3)	C20—C21—O3—C24	175.7 (4)
C11—C12—C13—C14	−179.4 (3)	C22—C21—O3—C24	−3.5 (6)
C7—C12—C13—C14	0.5 (4)	C7—N1—S1—O1	−48.0 (3)
C11—C12—C13—C15	5.1 (6)	C14—N1—S1—O1	166.9 (2)
C7—C12—C13—C15	−175.0 (3)	C7—N1—S1—O2	−176.6 (2)
C12—C13—C14—N1	−3.0 (3)	C14—N1—S1—O2	38.3 (3)
C15—C13—C14—N1	172.2 (3)	C7—N1—S1—C1	67.6 (3)
C12—C13—C14—C16	−177.1 (3)	C14—N1—S1—C1	−77.5 (3)
C15—C13—C14—C16	−1.8 (5)	C2—C1—S1—O1	−170.2 (3)
C13—C14—C16—C17	−45.6 (5)	C6—C1—S1—O1	13.1 (3)
N1—C14—C16—C17	141.1 (3)	C2—C1—S1—O2	−37.9 (3)
C14—C16—C17—C18	175.1 (3)	C6—C1—S1—O2	145.3 (3)
C16—C17—C18—C23	−3.2 (5)	C2—C1—S1—N1	76.3 (3)
C16—C17—C18—C19	177.6 (3)	C6—C1—S1—N1	−100.4 (3)

### Hydrogen-bond geometry (Å, º)

Cg2, Cg3 and Cg4 are the centroids of the C1–C6, C7–C12 and C18–C23 rings, respectively.

**Table:**

*D*—H···*A*	*D*—H	H···*A*	*D*···*A*	*D*—H···*A*
C6—H6···O2^i^	0.93	2.46	3.249 (5)	143
C15—H15*C*···*Cg*4^ii^	0.96	2.82	3.759 (4)	167
C24—H24*A*···*Cg*3^iii^	0.96	2.84	3.634 (6)	140
C24—H24*C*···*Cg*2^iii^	0.96	2.88	3.520 (5)	125

Symmetry codes: (i) *x*, −*y*, *z*−1/2; (ii) −*x*+1/2, *y*+3/2, −*z*−1/2; (iii) *x*, *y*, *z*+1.
