# Supplementary figures and images for: Crystal structure of (E)-2-(4-meth­oxy­styr­yl)-3-methyl-1-phenyl­sulfonyl-1H-indole
Source: Acta Crystallogr E Crystallogr Commun. 2015 Sep 12;71(Pt 10):o723–4. doi: 10.1107/S2056989015016631 (PMC4647445; doi:10.1107/S2056989015016631)

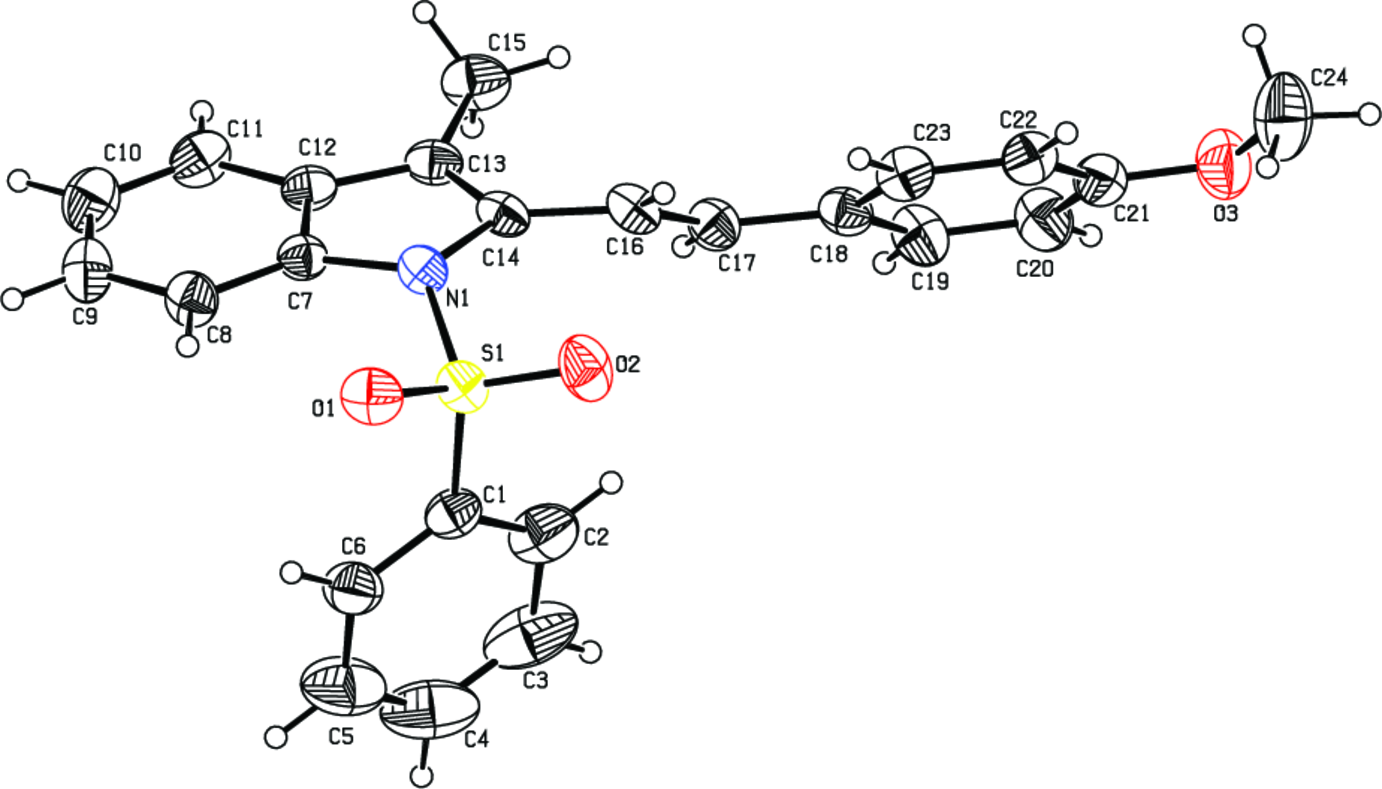

Supplement: Supplementary file 4 [file e-71-0o723-fig1.tif]

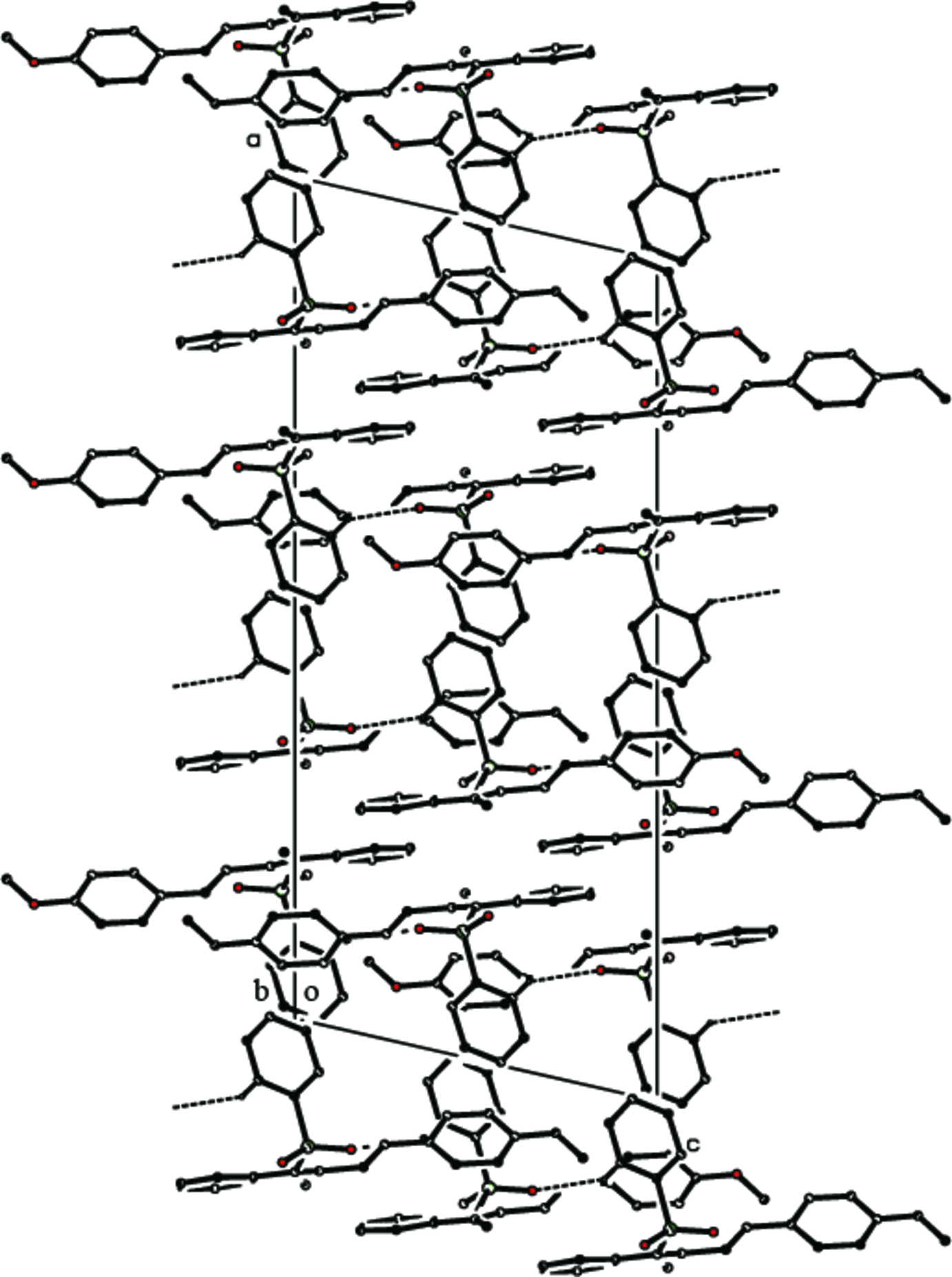

Supplement: Supplementary file 5 [file e-71-0o723-fig2.tif]
